# Measuring design diversity: A new application of Ostrom's rule types

**DOI:** 10.1111/psj.12440

**Published:** 2021-07-20

**Authors:** Claire A. Dunlop, Jonathan C. Kamkhaji, Claudio M. Radaelli, Gaia Taffoni

**Affiliations:** ^1^ Department of Politics University of Exeter Exeter UK; ^2^ Department of Management, Economics and Industrial Engineering Politecnico di Milano Milano Italy; ^3^ European University Institute, School of Transnational Governance Fiesole Italy

**Keywords:** institutional grammar tool, policy design, policy instruments, regulation

## Abstract

We draw on the Institutional Grammar Tool's rule types to empirically analyze the design of four major procedural regulatory instruments in the 27 member states of the European Union and the UK. They are: consultation, regulatory impact assessment, freedom of information, and the Ombudsman. By adopting the Institutional Grammar Tool as conceptual lens we end up with a single measurement template applicable to a variety of action situations. We derive measures that are conceptually robust and suitable for comparative analysis. With original data gathered on the official legal base in the 28 cases, we carry out principal components analysis. We identify design patterns across countries and instruments; the specialization of each instrument in terms of rule type; and the components that best explain cross‐country variation. In the conclusions we argue that to reframe the design features of the four instruments in conceptual, theoretical categories is not simply a taxonomical exercise but it extends to the territory of comparative policy analysis, practice and reform.

## 摘要

我们基于制度语法工具（Institutional Grammar Tool，简称 IGT）的规则类型，对欧盟27国和英国的四大程序监管工具设计进行了实证分析。它们分别是：咨询、监管影响评估（RIA）、信息自由（FOI）和欧盟监察专员。通过将IGT作为概念视角，我们提出了一个适用于不同行动场景的单一衡量样本。我们得出了概念健全、适用于比较分析的一系列方法。我们针对28国的官方法律依据收集了原始数据，并进行了主成分分析。我们识别了各国及监管工具的设计模式；每个工具在规则类型方面的专业化程度；以及最能解释跨国差异的主成分。我们的结论主张，从概念和理论类型对四大监管工具的设计特征进行重新建构，并不意味着简单的分类，而是扩展到比较政策分析、实践和改革领域。

## Resumen

Nos basamos en los tipos de reglas de la Herramienta de gramática institucional para analizar empíricamente el diseño de cuatro instrumentos regulatorios procesales principales en los 27 estados miembros de la Unión Europea y el Reino Unido. Ellos son: consulta, evaluación de impacto regulatorio (EIR), libertad de información (FOI) y el Defensor del Pueblo. Al adoptar el IGT como lente conceptual, terminamos con una plantilla de medición única aplicable a una variedad de situaciones de acción. Derivamos medidas que son conceptualmente sólidas y adecuadas para el análisis comparativo. Con datos originales recopilados sobre la base legal oficial en los 28 casos, realizamos análisis de componentes principales. Identificamos patrones de diseño en países e instrumentos; la especialización de cada instrumento en términos de tipo de regla; y los componentes que mejor explican la variación entre países. En las conclusiones argumentamos que replantear las características del diseño de los cuatro instrumentos en categorías conceptuales y teóricas no es simplemente un ejercicio taxonómico, sino que se extiende al territorio del análisis, la práctica y la reforma de políticas comparativas.

## INTRODUCTION

Sue Crawford and Elinor Ostrom's (1995) Institutional Grammar Tool (IGT) conceptualizes and makes operational the fundamental intuitions of the Institutional Analysis and Development framework. It provides a grammar based on the syntax of institutional statements and a set of rules differentiated by their semantics (Ostrom, 2005, p. 180). In the last decade, there has been a proliferation of studies informed by the IGT, often dedicated to the detailed examination of textual configurations found in policy documents and, in some rare cases, legislation. Most of the studies published so far are empirical and methodological, few are theoretical (for two recent reviews see Dunlop et al., 2019; Siddiki et al., 2020).

As shown by a recent literature review (Dunlop et al., 2019), IGT applications can be micro (analyses of statements included in institutional arrangements following Ostrom's institutional grammar ADICO [Attribute (A), Deontic (D), aIm (I), Condition (C), and Or else (O)]) or meso. Meso applications are not concerned with breaking down myriads of statements to their grammatical components but with the meso‐level constructs of typologies based on the classification of rules' aims. The micro approach is typically limited to one setting, be it one community, or one law. Studies within this approach are mostly computational and descriptive, and geared toward the identification and systematic grammatical analysis of large numbers of institutional statements. Applications at the meso‐level of rule types are quite rare though arguably more promising in terms of comparative analytical leverage (Dunlop et al., 2019; Espinosa, 2015).

Our analytical efforts are indeed focused on the meso approach which categorizes institutional rules into seven rule types—position, boundary, choice, aggregation, information, payoff, and scope. This typology is based on the different aims of institutional statements, standing as a generalization of ADICO (recall that “a*I*m” is one of the syntactic components of institutional statements) that allows for the reconstruction of action situations. In fact, “[r]ules are part of the underlying structure that constitute a single‐action situation or a series of them” (Ostrom, 2005, p. 179). In Ostrom's words, rule type categorization is “[…] a way of consistently grouping rules so that the analysis of rule systems can be made much more cumulative” and, we add, comparative (Ostrom, 2005, 175).

We bring in rule types to empirically analyze the structure and content of four major rulemaking instruments deployed by the 27 European Union (EU) governments and the UK. They are the following: consultation (notice and comment in US parlance), regulatory impact assessment (RIA), freedom of information (FOI), and the Ombudsman. These instruments lay down “rules to make rules” (see Radaelli, 2010a, 2010b on meta‐regulation). We focus on these four specifically, rather than, say, inter‐institutional arrangements or guidance on legislative drafting, because they are *relational* instruments that bring in and enfranchise stakeholders, experts, and the public at large. This focus on rule types enables us to capture and compare the structure and relational attributes of the four action situations generated by each instrument.

We contribute to the IGT meso‐level applications by showing how it can be applied to a relatively large number of cases, that is four rulemaking instruments across 28 countries. Considering that each instrument is anchored to a legal base comprising laws, regulations and guidance documents, the empirical base is vast. Our choice to work with rule types over ADICO (for more see Introduction to this special issue) reflects the limitations of conducting a syntactic analysis of four sets of legislation and/or guidelines for procedures in 28 countries in more than 20 languages that goes beyond computing the frequencies of ADICO components.

We also contribute to literature on policy designs (Carter et al., 2016; Siddiki et al., 2011) drawing on the IGT to make comparisons at three levels: rule types, instruments, and countries. We identify design patterns across countries and instruments; the “specialization” of each instrument in terms of rule type; and the components that best explain cross‐country variation. This IGT‐inspired contribution on regulation is original. No study has examined these instruments and procedures together (with or without the IGT). We demonstrate the agility and adaptability of the IGT to different research questions, bringing the analysis to a level of granularity that is not achieved by conventional propositions about families of countries and legal traditions.

This takes us to our final contribution. Exactly because we adopt the IGT as the conceptual lens to view a variety of action situations, we are able to derive measures that are conceptually robust and suitable for the comparative analysis of policy designs. The categorization of rules benefits policy analysis at large, that is, beyond the empirical realms where they were first conceptualized and deployed. This is because rule types draw on the universality of language (including, clearly, institutional language) and meaning to reduce and organize the virtually infinite empirical diversity and complexity that characterizes institutional action.

To illustrate: regardless of how rules regulating the access to common pool resources or to consultation procedures manifest themselves empirically, conceptualizing them as boundary rules enables useful comparisons not of the rule themselves^1^ but of the action situations they shape and of the overall rule configuration within action situations. As such, empirical applications are potentially limitless. For the policy analyst, rule types have the same guiding function as anatomy books for medical doctors. Every patient has two knees, the anatomy book describes knees in general so that a medical doctor can recognize them across a vast array of patients (action situations), although no knees are empirically the same. Yet, the recognition of functional similarity within diversity allows for diagnosing the pathologies and the success of the overall configurations.

In the remainder, we present: (a) the motivation behind our approach, (b) the research questions, (c) the original data used to address them, and (d) our analysis. We conclude with reflections on the potential of our analysis and how it can be exploited. We argue that to reframe the design features of the four instruments in conceptual, theoretical categories is not simply a taxonomical exercise but it extends to the territory of practice and reform.

## PROCEDURAL REGULATORY INSTRUMENTS AS ACTION SITUATIONS

For scholars of policy design, regulatory‐procedural policy instruments present three formidable problems. First, there is the challenge of concept formation. How does our choice of a concept match the selection of the policy instruments? For more than two decades, international organizations and governments have promoted the so‐called “better regulation” agenda (on the EU see Listorti et al., 2019; OECD, 2019). Social scientists have studied the adoption, instruments, implementation, and indicators of this agenda (Bozzini & Smismans, 2016; Radaelli, 2005, 2020; Wiener, 2007). Yet, the conceptual extension and intension of “better regulation” do not necessarily match social scientific standards. Concept formation should come from a theoretical (as opposed to descriptive) approach. We cannot expect international organizations to do this.

The IGT allows us to go beyond the labels of our four instruments. By putting them under the same theoretical lens (IGT's rule types), we “see” their empirical manifestations as designs of four distinct but interrelated action situations. The nature of this interrelation lies in what these procedural action situations do: they constrain governments and regulators and hence enfranchise public interests in public policy‐making. More precisely, these constraints and obligations stipulate that: new primary or secondary legislation require a process of consultation and an impact assessment of the proposal; individuals can obtain access to the process via FOI requests; and, finally, the Ombudsman can represent interests potentially infringed by an administrative decision.

After concept formation we have to tackle measurement. To measure policy designs we need indicators. International organizations such as the World Bank and the Organization for Economic Cooperation and Development (OECD) provide cross‐country regulatory performance measures (https://rulemaking.worldbank.org/; OECD, 2019). There are also regulatory indicators produced by political scientists, notably the effort to capture density and intensity of regulation (Knill et al., 2012). Measurement should be aligned with concept formation and theory. It should not be sensitive to the idiosyncratic characteristics of the individual instrument or political system. The World Bank and OECD indicators of rulemaking are mainly descriptive.

The final problem concerns research design. How can we compare the different design architectures? How can we dissect their structure and relationships? The solution to these problems starts from a transparent definition of what we put into the box of “regulatory procedural instruments,” or “better regulation.” Different choices are legitimate, including the choices made by international organizations that have promoted the “better regulation” agenda. Our choice is not to use “better regulation” because it is a value‐laden category and does not usually include access to information and the Ombudsman. We identify conceptually the policy instruments that define action situations that have a family resemblance (Maggetti et al., 2012, Chapter 2). They are not nested (although consultation is often included in impact assessment), but they share the above‐mentioned properties of constraining bureaucratic action and enfranchising diffused interests through participation and transparency.

Let us take a closer look at the four instruments.

The aim of consultation is to bring evidence to bear at the stage of policy formulation (yet again, primary and/or secondary legislation, depending on the country). It pushes governments and regulators to take into account the evidence‐based input from stakeholders and citizens. There is significant variation on how consultation is carried out in the EU (Bunea, 2016) and across its member states. A first difference is whether consultation happens at an early or later stage in the development of laws and regulations. The latter is more common in the EU (OECD, 2019). There is also cross‐country variation about whether consultation is mandatory or not. There are in fact European countries in which public bodies have close informal contacts with specialized interests. Hearings have characterized the policy process of Northern countries for a long time. In others, formal consultation procedures appeared in the last 25 years, with the diffusion of the OECD principles of regulatory quality and the emergence of an EU‐policy in this domain (De Francesco, 2013).

RIA is an evidence‐based procedure that prescribes a set of tests and analyses on proposed legislation (primary and/or secondary). RIA informs on how stakeholders are likely to be affected by the policy proposal under consideration. In some cases, governments and regulators must also report the likely impacts on the environment, trade, human rights, and gender. Thus, RIA is an integrated procedure involving different analytical and consultive steps. Once these procedural steps of RIA are carried out, policy problems and objectives, data, and expert and stakeholder opinions are coalesced in a single document. Out of 28, 26 countries have a formal requirement to conduct a RIA to inform the development of primary legislation. Among the 28 cases, 16 require RIA for secondary legislation (OECD, 2019). In the USA, by contrast, only regulations proposed by federal executive agencies are subject to impact assessment. Further, while t in Canada and the USA RIA revolves around varieties of cost‐benefit analysis, the European approach is more diverse and cost‐benefit analysis sits alongside other techniques. Finally, in the USA the RIA is published for notice and comment. In the EU, instead, there is more variety (on transatlantic differences, see Strauss et al., 2008).

FOI procedures are our third action situation. FOI is widely recognized across the world as a hallmark of transparent government (indeed, each year there is an International Day for Universal Access to Information [IDUAI; Unesco, 2016] to further promote the FOI cause). Dating back over 250 years, over half of the worlds' FOI laws have been adopted in the past two decades—a trend that has been stimulated by pressure from civil society, international and donor organizations (Banisar, 2006; Mendel, 2008; UNESCO, 2016). Europe serves as a microcosm of this global diffusion—with first adoptions in Western Europe in the 1970s followed by a second wave from the CEE EU accession countries in the 1990s. Despite all 28 cases being covered in some way, there is an important variation on multiple dimensions. Most notably, these include the bodies covered, documents and/or information which is accessible, exemptions allowed for information types, fees to be charged, timescales that must be followed; appeals process, oversight and investigation arrangements, and sanctions for refusal to grant information.

Finally, comes the Ombudsman. The term comes from Scandinavian languages and indicates a person who works as a representative, an agent. The Ombudsman serves the diffuse interests in the polity by acting on behalf of these interests before administrative bodies in cases of maladministration and injustice. Early forms of this institution are documented since the 12th century but it is conventionally assumed that it took its modern form in Sweden at the beginning of the 19th century. Since then, different waves appeared (Gregory & Giddings, 2000). In the first wave, the Ombudsman spread across Scandinavia in early 20th century. But it is only in the post‐war period, under the second and third waves, that the institution started its global diffusion, first across Western democracies and then across new democracies. According to the International Ombudsman Institute,^2^ 140 countries in the world have established an Ombudsman office.

When it comes to functions and mandate, the Ombudsman is an officer of the legislature vested with broad supervisory and investigative powers and jurisdiction over the public administration. As such, it is thought to promote accountability and protection of individual rights, ultimately fostering rule of law and democracy (Diamandouros, 2006). Key features of the office are independence, broad accessibility, vast latitude in terms of investigative powers, a largely informal role when it comes to the range of remedies, which typically take the form of mediations/conciliations between the parties or recommendations issued to the investigated public authorities.

The Ombudsman usually lacks means to enforce its recommendations and issue sanctions. It follows that the implementation of Ombudsman's recommendations mainly depends on its moral suasion and the levels of trust and social capital in society. In Central and Eastern European countries the type and bite of the Ombudsman's remedies tend to be broader. Although barred from issuing sanctions on its own, the system of referrals of cases to judiciary or disciplinarian bodies strengthens the cogency of Ombudsman recommendations, making the office closer to courts.^3^

These four instruments have a common procedural nature. They start when an actor performs a given action, others respond, there are interactions, exchanges of information, choices and appeals, until, ultimately, an end point is reached and a decision made. By adopting the IGT, we conceptualize the four instruments as action situations where policy actors: inhabit roles, have rights, make choices, lodge complaints, and face consequences while drawing on information they access.

We therefore argue that nationwide, whole‐of‐government rulemaking procedures in a population of countries can be approached as empirical instances of rule types that constitute an action situation. We adopt the rule categorization aspect of IGT specifically. According to Ostrom, rule types are a classification instrument, “a useful system for those interested in linking rules and the action situations (games) created by rules, the biophysical world, and communities” (Ostrom, 2005, p. 187). We postulate therefore that the design of a rulemaking procedure such as consultation or impact assessment constitute the sufficient set of rules‐in‐form that shapes the “consultation/RIA action situations.” “Rules are part of the underlying structure that constitute a single‐action situation or a series of them” (Ostrom, 2005, p. 179).

We further argue that the four selected procedures/instruments share the characteristic of opening‐up the public decision‐making process to a range of interests. In other words, they share the property of constraining bureaucratic action and enfranchising diffused interests through participation and transparency. The unifying element of these procedures, and what makes them shape up the regulatory policy process, is the sheer similarity we observe, across countries and procedures, in position rules (and the boundary rules which qualify them). In fact, along with highly certified bureaucratic actors like the drafting authority of consultation/IA, the information commissioner (IC) of FOI procedures or the Ombudsman, these participatory rulemaking procedures systematically assign a position to either citizens at large or qualified non‐bureaucratic actors such as experts (in RIA), stakeholders (in consultation), or affected parties (in FOI and OM procedures).

Thus, the significance of the rule types lies in their role as markers of important steps when actors interact, become interdependent, or are requested to perform in an institutional setting (Schlager & Cox, 2017). These interactions can be understood as comprised of the IGT seven rule types—position, boundary, choice, aggregation, information, payoff, and scope (Ostrom, 2005, 2007, pp. 29–30; see Box 1). Each of these seven types has a different manifestation in each of the four instruments (see Table 1 for exemplars). Empirically, our aim is to explore the balance of these seven types in each instrument across 28 countries. Since we are interested in design, we look at rules‐in‐form only.

**TABLE 1:** Seven rules as administrative tools: exemplars

	Position	Boundary	Choice	Aggregation	Information	Payoff	Scope [Promotion of …]
Consultation	Departments	Definition of who can take part in consultation	Dept. has to set a timetable	Institutional settings for checking the quality of consultation	Publish annual plan for consultation	Dept. has to motivate why some concerns were not accepted	Engagement
	Independent regulators	Time boundaries	Procedural steps to seek the views of experts		Publish an invitation to parties to submit comments		Inclusiveness
	Stakeholders		Range of consultation techniques				Non‐discrimination
	Regulatory oversight body						Plain language
Regulatory impact assessment	Departments	Exceptions	Requirement to evidence the presence of market failure	Conditions under which actors have to agree collectively on RIA (e.g., cabinet meetings)	Publication of draft RIA	Depts must revise RIA if it does not meet quality standards	Transparency
	Independent regulators	Mandate of the oversight body	Analysis of administrative burdens		Publication of opinions of the regulatory oversight body		Quality of business environment
	Regulatory oversight body	Who qualifies as expert	Benefit‐cost criteria				Gender equality
	Experts						Sustainablity
Freedom of information	Requestor	Definition requestor eligibility (e.g., include non‐citizens?)	Actions possible for all three positions with concerning what can be:	Provisions in place for consultation with third parties that may be relevant to information requests	Information/documentation formats	Redress procedures for underperformance	Promotion of best practice
	Information Commissioner	Public authorities that are excluded (e.g., Ministry of Defense)	requested withheld/refused appealed investigated		Timescales for information release or appeal	Sanctions for unsupported restriction of access or defacement/destruction of information/documentation	

Dedicated appeal body				Fees for information release or appeal		
Ombudsman	Ombudsman;	Personal interest of the complainant;	Investigations	Referrals to judiciary, disciplianarian or legislative bodies	Obligations to share information with the Ombudsman	Sanctions (administered by a third party)	Accountability
	Complainant;	Time boundaries;	Remedies (mediation or recommendations)				Individual rights Good administration
	Public body under investigation;	Range of bodies under OM's jurisdiction;					
	Other public bodies recipient of referrals	Incompatibility with judicial procedures

BOX 1 **Seven rules types**

**Table:** 

Rule type	These rules …
Position	Identify positions/roles to be filled by individual or collective actors
Boundary	Regulate eligibility of actors to occupy positions
Choice	Specify actions that actors must, must not, or may undertake
Aggregation	Discipline actions or decisions that require the aggregation of two or more actors
Information	Identify channels and modes of communication/exchange of information between actors
Payoff	Assign benefits and costs—for example rewards and sanctions—to specific actors relative to distinct courses of action
Scope	Identify required, desired, or prohibited outcomes of the action situation

*Source:* Carter et al. (2015, p. 163), Ostrom (2005, p. 190).

## DATA AND RESEARCH QUESTIONS

Empirically, we find the rule types that constitute the design of the action situations in the legal base of the instruments and procedures. The IGT is eminently suitable for the collection of data and analysis of formal laws (Ostrom, 2011, p. 18). Our legal base comprises laws (in the case of FOI and the Ombudsman) and guidance documents. In some countries RIA and consultation are not established by law, yet governments publish official guidance on how they should be carried out by officers in departments and agencies. Our “four‐action‐situations” strategy is consistent with the IGT literature that has examined networks of adjacent (McGinnis, 2011) and layered action situations (Möck et al., 2020).

To achieve the typological form of measuring implied by IGT's rule types, we worked with a team of 40 administrative lawyers. For each country, we identified the legal bases of the four instruments in force as of June 2018 (grounded in hard law or on soft guidance documents) and retrieved text in original language and in English translation. Relevant portions of legal texts were gathered using a protocol based on Ostrom's rule types. Thus, when considering the guidance and/or law on consultation for country X we retrieved the exact text (articles, clauses, or entire sections) where positions are defined, boundaries set, information flows described, choice prescribed, and so on. Data collection was completed in December 2018, when the UK was still a member state of the EU.^4^

With these data, we address the following research questions about the rule structures and action situations we examine in the context of our population.^5^

*RQ1* How do the four action situations differ in terms of rule types? To answer this question we take the 28 countries as a single population containing 203 rules, distributed by IGT type.

*RQ2* What are the features of the rules that best explain the variation in our population? The IGT provides a powerful and theoretically robust lens to observe the fine‐grained (i.e., at the individual rule type level) variability of procedures. But, we are interested in going one step beyond simply mapping variation overall. Using Principal Component Analysis (PCA) we uncover the key difference‐making components of each instrument.

*RQ3* Having identified the components that matter in explaining variation, we ask: how do countries align in relation to the components that best explain variation?

## ANALYSIS

We begin our analysis considering the overall distribution of the rules by IGT types across the four instruments, ignoring for the moment country‐level variation (Table 2).

**TABLE 2:** Rule numbers by instrument

	Position	Boundary	Choice	Aggregation	Information	Payoff	Scope	Total
Consultation	7	4	8	0	6	0	8	33
	21.21%	12.12%	24.24%	0	18.18%	0	24.24%	100%
RIA	10	7	17	0	4	1	6	45
	22.22%	15.56%	37.78%	0	8.89%	2.22%	13.33%	100%
FOIA	3	23	16	1	12	5	4	64
	4.69%	35.94%	25%	1.56%	18.75%	7.81%	6.25%	100%
OM	2	17	26	3	8	4	1	61
	3.28%	27.87%	40.98%	4.92%	13.11%	6.56%	1.64%	100%

The IGT sheds light on the structure of rules across the four instruments. Payoff and aggregation rules are rare in all cases. This points to limited reach in terms of scrutiny, oversight, sanctions, and rewards. The incentive structure is not based on tangible implications for government departments and agencies of not performing according to guidance or rewards for good practice. For example, the only aggregation moment for FOI concerns specialist cases of consultations with third parties when dealing with information that may impact adversely on them.

For the rest of the rule types, the picture is mixed. Position rules are a case in point. The design of FOI and Ombudsman displays conventional positions that determine who can participate. Take FOI: four positions recur: (a) the requestor (usually the public in some form), (b) a public authority, (c) a specialized information appellate body—usually called the IC, and (d) a designated information handler that sometimes exists in the bureau or within each public authority. The Ombudsman is similar with three clearly codified positions (the Ombudsman, the complainant, and the investigated public body). The degree of codification is lighter in RIA and consultation.

The position of who carries out the RIA ranges from the “individual officer,” the “competent administration” (Estonia, Lithuania, Italy), the “initiator of the act or external contractor” (Romania) to more generic references to decision‐making in cabinet (Spain). Specific RIA positions are sometimes assigned to Treasury (control on the costs of proposed legislation), the Ministry of Justice (control on the quality of legislation), the legal service (Cyprus), and independent regulators. In consultation, there is no identification of who exactly carries out the procedure in a number of countries, including Austria, the Czech Republic, and Denmark. By contrast, countries like Bulgaria define the position of “the drafting authority” with some precision—this authority can be a central government department or an independent regulator. In federal countries, position rules include sub‐national authorities.

FOI and the Ombudsman are heavy on boundary rules, whereas consultation and RIA set fewer barriers. Indeed, with FOI and Ombudsman we enter a world of conditions, exceptions, and exemptions where definite eligibility criteria are attached to each of the positions. In the case of FOI, for example, these rules offer precision on who can request and what constitutes a public authority and an IC. We also find the boundaries of the information and/or documents themselves. This is one of the central dimensions where FOI vary across the world—so‐called class and harm tests. In essence, these cover the exemptions—which can be either mandatory or discretionary—to particular categories of information (class) or information whose release is judged to risk harm to certain functions of the state. In Ombudsman procedures, boundaries to eligibility similarly apply to the complainant, in the form of demonstrating a personal interest/suffered violation and of filing the complaint within a specified time‐frame (typically one year), and to the public administration, in the form of exempted bodies.

Choice rules feature strongly in all four instruments. For RIA and consultation, these rules refer to the steps of the procedures. These are mostly procedural‐analytical steps and tests in RIA, such as measuring the baseline and examining more than one option. In consultation, choice rules deal with identification of parties, notification, consultation timetable, and other steps, including in some cases (e.g., Bulgaria) seeking experts' opinions. In FOI, for requestors, obligations, and rights revolve around information re‐use and appeals. For public authorities, disclosure actions, rules of engagement with the requestor, reporting requirements, and obligations in the appeals process all have prominence here. Where an ICO exists, choice rules concern the nature of their decision‐making—binding or not—the extent of their powers and, again, reporting activities. In the Ombudsman, where we see the greatest number (41%), they mainly reflect two key aspects of the procedure. First, the investigative functions of the Ombudsman which trigger the relational aspect of the action situation. Second, the overarching dimension of remedies. Indeed, the accountability potential of the Ombudsman is muted lacking clear rules which discipline the means through which cases of maladministration or violation of individual rights can be mended. This is also the reason why the Ombudsman is comparatively the instrument featuring most aggregation and payoff rules (although few).

As we would expect from an information tool, FOI information rules are abundant. They cover a vast range of details regarding the timing, format, record management procedures, and the clarity of the process. But all procedures contemplate information rules, given that they are contingent on increasing transparency, notifying, giving reasons, and displaying evidence utilized by the government and the regulators.

Finally, turning to scope rules, statements on the overall aims and outcomes to be achieved are scarce in instruments grounded in codified law—that is, FOI and Ombudsman. Discussions about the scope of the instrument are found in the legislative and political debates that pre‐date the instruments' design and enactment. For consultation and RIA, the picture on scope rules is different. As instruments set in guidelines rather than law, motivations and aims are recorded to underline their importance. Consultation in particular is the instrument through which governments send signals and generate expectations about the involvement of a range of interests and preferences that by design are enfranchised. In contrast to other ways of influencing the legislator or rule‐maker, consultation is where the legal base provides for access to draft rules of “any citizen,” “interests not directly affected” and “citizens of other countries that may be affected” (this wording occurs in the legal base). This is also the procedure with the lowest number of rules, which signals the presence of degrees of freedom in how to carry out consultation as well as reflecting the fact that consultation guidance is generally short and, in many cases, embedded in the RIA procedure.

We now examine the key dimensions of variation in each policy instrument, commencing with consultation (Table 3). We do this through four principal component analyses (PCAs), one for each instrument. PCA is an exploratory dimension reduction technique which allows for the reduction of redundant information and the identification of principal components. The latter are computed as orthogonal linear transformations of the original manifest variables and are used to reveal a simpler internal structure of the data. The type of PCA we have employed maximize the variance in the data as we want our principal components to represent those dimensions that most explain variation across our cases. We discuss our approach and the individual components of each instruments (and what they mean) in the Appendix.

**TABLE 3:** Principal component analysis: consultation, RIA, FOI, and Ombudsman

Consultation				Regulatory impact assessment			
Principal components	Share of explained variance (cumulative)	Loading variables and coefficients	Type of rule	Principal components	Share of explained variance (cumulative)	Loading variables and coefficients	Type of rule
1) Commitment	28.2%	Is there a generally applicable, nationwide, cross‐cutting legal base for consultation? (0.901) Does the Drafting Authority (DA) have to set a consultation timetable? (0.875) Does the DA have to publish a report on comments filed by the CEs (consultation report)? (0.888)	Background Choice Information	1) Breadth of exceptions	27%	International treaties, Constitution, EU and for fed countries regulations concerning multi‐level governance (0.878) regulations with a mere formal nature and self‐regulation of the government (0.816) Urgency (0.815) State budget (0.705)	Boundary Boundary Boundary Boundary
2) Scope	25.9% (54.1%)	Does the legal base spell out inclusiveness of groups that may not be directly affected as aim of the consultation procedure? (0.782) Does the legal base spell out avoiding discrimination as aim of the consultation procedure? (0.917) Does the legal base spell out understanding via plain language as aim of the consultation procedure? (0.862)	Scope Scope Scope	2) Analysis	15.5% (42.5%)	Does the legal base contain requirements to analyze the status quo? (0.912) Does the legal base contain a requirement to compare or identify or commensurate benefits and costs? (0.793)	Choice Choice
				3) Publicity	13.2% (55.7%)	Does the legal base mention line departments (as drafting authorities)? (0.872) Are draft RIAs published? (0.832)	Position Information

Freedom of information				Ombudsman			
Principal components	Share of explained variance (cumulative)	Loading variables and coefficients	Type of rule	Principal components	Share of explained variance (cumulative)	Loading variables and coefficients	Type of rule
1) Information commissioner: presence, powers and paperwork	22%	Presence/absence of dedicated information commissioner (0.804) Are information commissioner decisions binding? (0.796) Does the information commissioner have inspection powers? (0.867) Can the information review classified documents? (0.907) Must the information commissioner report to legislature? (0.889) Is there a documented appeal process in the legislation? (0.859) Is there a clear timeline for appeal in the legislation? (0.821) Does the legislation require the sharing of best practice by a dedicated body? (0.727)	Position Choice Choice Choice Choice Information Information Scope	1) Remedies	27.7%	Can the OM issue binding recommendations? (0.797) Upon receiving an OM recommendation, is there a specific deadline for the concerned public body to comply? (0.924) Is the concerned public body obliged to comply with the OM's recommendations by notifying her about actions taken? (0.939)	Choice Choice Information
2) Boundaries of discretionary harm tests	18% (40%)	Does the legal base give government discretion to deny information that could cause harm to persons? (0.890) Does the legal base give government discretion to deny information that could cause harm to international relations and defence? (0.968) Does the legal base give government discretion to deny information that could cause harm to commercial competitiveness? (0.890) Does the legal base give government discretion to deny information that could cause harm to national economic interests? (0.871) Does the legal base give government discretion to deny information that could cause harm to the activities of law enforcement agencies? (0.968)	Boundary Boundary Boundary Boundary Boundary	2) Breadth of accountability	18.7% (46.4%)	Does the legal base put private entities performing public functions under the OM's jurisdiction? (0.879) Is the periodic report to the body which appoints the OM public? (0.875)	Boundary Choice
3) Boundaries of mandatory and discretionary class tests	12% (52%)	Does the legal base contain a mandatory class test on information and documents pertaining to national security? (0.775) Does the legal base include a mandatory class test on information and documents pertaining to national economic competitiveness? (0.761) Does the legal base contain discretionary class tests on information and documents pertaining to national security? (−0.874) Does the legal base contain discretionary class tests on information and documents pertaining to personal information? (−0.843)	Boundary Boundary Boundary Boundary	3) (Ecological) boundaries	14.3% (60.7%)	Does the complainant have to hold a personal interest to be allowed to file a complaint? (0.834) Does an ongoing judicial procedure prevent the OM from launching an investigation? (0.849)	Boundary Boundary

Variation in consultation designs is driven by two components that capture fundamental features of this instrument as well as the importance of certain types of rules (Table 3). PC1 is about commitment. We identify a first “background rule” that is not captured by Ostrom's type but is essential in our case, because we are dealing with two different approaches to consultation. Some countries follow the formal approach, based on provisions contained in either hard law or soft law, or in some cases both. These provisions describe the steps and actions of the government during consultation, no matter what sector is considered. The other set of countries comprises the cases of informality as guiding principle for consultation and cases where there is no consultation.

The second rule of PC1 is an IGT choice that commits the departments or agencies to the production of a timetable at the beginning of each consultation. Some countries do not have a rule‐type like this because the timetable is uniform for all consultations and fixed by law or government's decision (UK). Others do not have the timetable rule because departments and agencies organize consultation with some flexibility and informality (Sweden). The third rule is about the provision of information relevant for the overall credibility of the exercise, that is, the drafting authority publishes a report at the end of consultation showing how the comments raised by the stakeholders were taken into consideration. Together, these three rules signal the commitment of the government to consultation—hence the label of this component. In IGT language, commitment is a combination of uniform cross‐sector standards that create expectations about the process, choice, and information.

PC2 is straightforward: it is about the IGT category of scope. We find three scope rules. They open up consultation to interests that otherwise would not be considered—the interests of those who are not directly affected, who would be discriminated, and who would not understand draft legislation because of technical language. We said earlier on that the design of consultation is among other things a signal. To employ words like the ones captured by the three scope rules sends a signal of openness and non‐discrimination.

**FIGURE 1:**
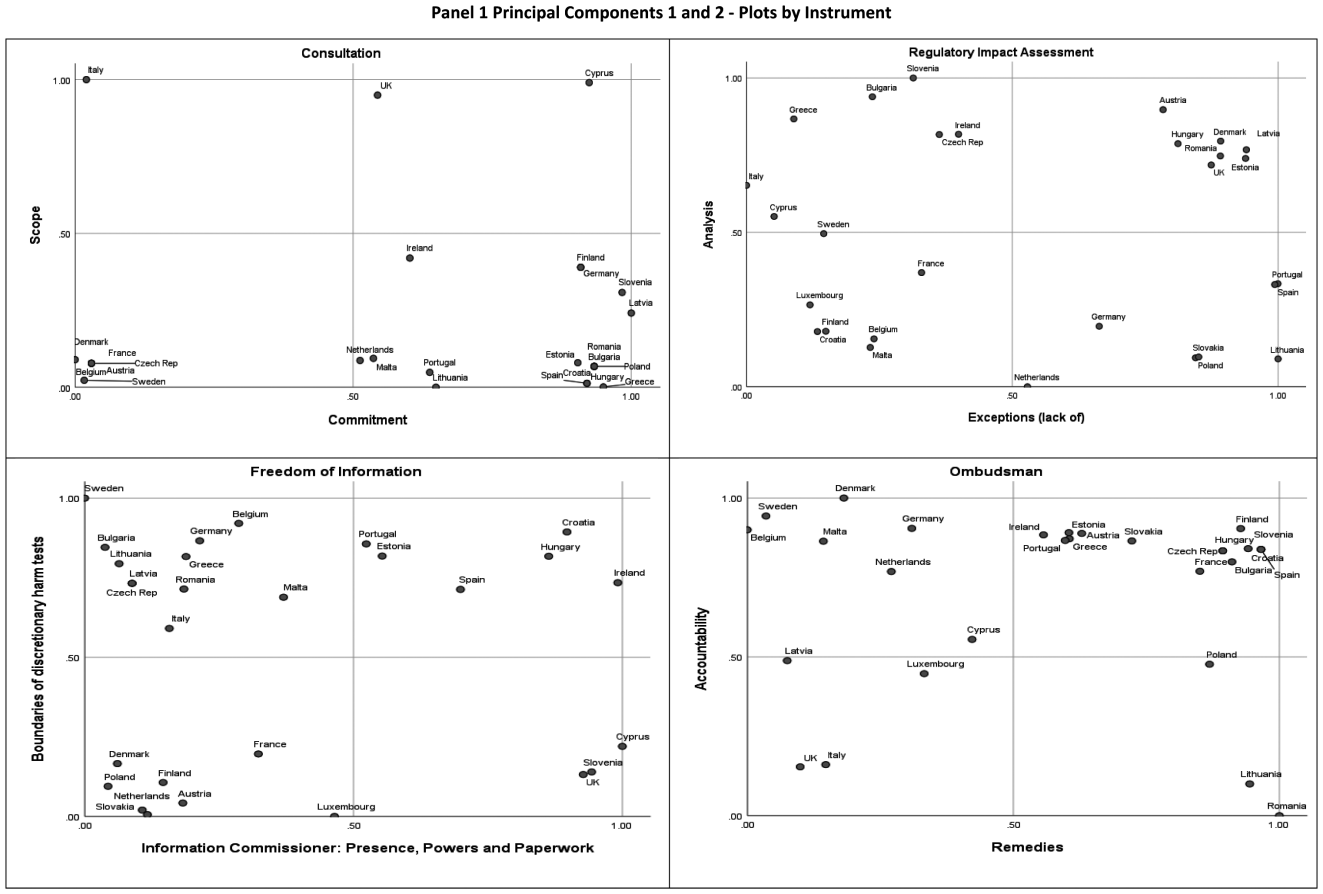
Principal components 1 and 2—plots by instrument

The distribution of countries on the two components is portrayed in the X/Y plot in Figure 1.

We find that most countries cluster on the lower quadrants, meaning that few countries invest in scope rules. Italy, Cyprus, and the UK are in the upper part of the figure, but in different positions in relation to PC2. The case of Italy is one of investment in scope rules but not in commitment. We suggest this is indicative of a flowery language without specific obligations (Italy is low on PC2). The Republic of Cyprus has high scores on both scope and commitment. The legal base for consultation is indeed one of the richest we have found in our population—it contains an obligation to write thank you letters to those who have taken part in the exercise. This over‐presence of rules, when confronted with the practice in Cyprus (OECD, 2019, Chapter 2; personal communications with our Project Expert for Cyprus, May 2020) suggests “communication out of character.” The legal base communicates an idealistic consultation that is at odds with reality.

In the lower part of the figure and to the east we find countries that have historically championed informal consultation, quite different from “better regulation” OECD‐style (Dunlop et al., 2020) either because of informality (Denmark, see Radaelli, 2010b) or corporatism (Austria). Sweden has its own approach—based on delegation of consultation on secondary legislation to regulatory authorities and, for primary legislation, consultation via committees of inquiry and hearings. This approach does not contemplate the steps and formalities presupposed by the typical OECD and EU practice (Radaelli, 2009, 2010b).

The south‐west quadrant displays 12 countries that have high scores of commitment and low values on scope. We read this first of all as evidence of convergence across systems that do not share historical traits in terms of diffusion of administrative law or membership of the EU—both a founding member of the EU (Germany) and the most recent entrant (Croatia) are in this group. Consultation as portrayed in the map does not follow any conventional narrative. The countries are not displayed in ways that resemble our knowledge of pressure group systems (social dialog and corporatism versus pluralism) and old‐new member states of the EU. And second, the map points to a prevalence of commitment over declaratory functions of the legal base.

We now examine the components of RIA (Table 3 and the X/Y plot on RIA in Figure 1). PC1 has a clearly discernible IGT value: it is about the boundaries. It is made up of four types of exceptions—cases in which the government does not have to carry out an impact assessment. Exceptions are the most important boundary rule. There are few boundaries of the “who” of impact assessment—generally the position rule is the line department. Instead, the boundaries concern the “what,” that is, the RIA.

As for PC2, it gathers two analytical requirements. RIA as procedure requires departments to undertake a range of tests and analyses, from the initial identification of the status quo to the comparison of alternative feasible options. Our component assembles the initial test on the status quo—which in the legal base is sometimes described as identification of the current regulatory‐legal base, or definition of the problem that needs to be addressed—and the benefit‐cost principle or criterion. In our population, the latter is not formal benefit‐cost analysis, but rather a requirement to commensurate positive and negative impacts, or take into consideration some categories of qualitative benefits, or justify costs with the benefits accruing from the chosen option (or from a range of feasible options). The IGT lens shows the nature of choice rules of PC2.

PC3, which we label “responsibility” is about “who” carries out the RIA—in some cases the legal base is silent, assuming that RIA shall be done, but without clarifying who exactly will take responsibility. The publication of draft RIAs is another step that points toward responsibility. Some governments do not publish RIA because either do not have this instrument (except that in special sectors like the environment or for a type of companies, such as Malta with the Small Business Act) or because the process of appraising the likely effects of proposals is not formal. In IGT terms, PC3 is a combination of position and information rules.

Turning to the distribution of countries portrayed in the second X/Y plot in Figure 1, the design of RIA is analytical (PC2) and carried out across the board (PC1) in seven countries that differ by administrative traditions and experience. Our communications with the Project Experts in Romania and Hungary point to the likelihood of “communication out of character.” The legal base was inspired by the OECD‐EU principles of regulatory reform; however, there are administrative capacity issues on the ground in these two countries (World Bank, 2015; Personal communications with Project Experts February 2020). In the north‐west quadrant, the countries are more distanced. Here RIA is not carried out across the board, but when it is done it is comprehensive. Boundaries may then signal sector‐level meticulous guidelines (e.g., the gas and electricity sectors in Italy).

The south‐west quadrant signals low density of IGT rules (at least as far as the first two components are portrayed). The boundaries are low because the RIA procedure is barely sketched (Belgium, Malta, Luxembourg) and because there is a preference for rules‐in‐use rather than rules‐in‐form (Finland).

Variation in FOI legislation is accounted for by two features—the presence, powers, and paperwork associated with a dedicated independent supervisory body (IC) that may exist as an audience for appeals and the boundaries concerning what documents and/or information can exempted from disclosure.

PC1 comprises eight variables revealing the pivotal importance of the IC position. Central to the IC operation are the choice rules attached to that role and specifically, whether: its decisions are binding; it has inspection powers; and it must report annually to the legislature. Added to this, information rules concerning the existence of a delineated appeal process and timeline account for diversity. Finally, we have a single‐scope rule usually associated with the IC as an engine for the sharing of best practice. In short, this is a microcosmic action situation concerning the operation (or not) of a dedicated FOI appeals that process within the overall instrumentation.

PC2 and PC3 underline the importance of the presence or absence clauses in the law that exempt information—harm and class tests. FOI legislation contains an array of these tests, but this analysis cuts through the complexity. PC2 shows that one set of boundaries that matter is the absence of discretionary harm tests across five main categories (Blanke & Perlingeiro, 2018, pp. 33–38; Muscar & Cottier, 2017; OECD, 2011). These are, harm to persons, international relations, commercial competitiveness, economic interests, and the activities of law enforcement agencies. PC3 similarly focuses attention on the boundary rules that dominate FOI. This time we are dealing with the presence or absence of tests around entire classifications of information and whether these are mandatory (in relation to national security and economic competitiveness) and discretionary (in relation to national security, personal data and commercial confidentiality).

The data suggest that the mix of other rules—choice, information, and scope—matters *but* only as they relate to the presence or absence of one position—the IC. Despite the fact that scope rules account for only 6% of the FOI content analyzed and are rare in this policy instrument, they do matter in connection to the presence of an IC to operationalize them. And, while information rules are in abundance in FOI legislation (accounting for nearly a fifth of our data structure) they do not drive cross‐national variation even though there is a good deal of diversity in these rules across the 28 cases. Rather, their importance relates only to the issue of appeals.

Staying on the theme of surprises, when we consider the legal literature on FOI legislative design, there are some variables that are assumed to make a difference between countries but just do not figure here (despite considerable cross‐national variation). These include: whether the legal text gives requestors access to both information and specific administrative documents (Dragos et al., 2019); the presence of a so‐called public interest over‐ride invoked as a final check before exceptions are applied (Banisar, 2006); the presence of fees for information access (Banisar, 2006); the sanctions imposed for violations of FOI legislation (Blanke & Perlingeiro, 2018, pp. 58–60).

When we look at the two main FOI components, the 28 cases fall into distinct zones (FOI X/Y plot in Figure 1). Taking the bird's eye view, as we move eastward we encounter countries with an IC whose powers are considerable and with an appeal process whose rules are clear (the ideal type being the UK^6^). As we move northward, we find fewer and eventually no discretionary harm tests (the archetype being Sweden). The north‐west quadrant contains the largest concentration of countries—nine in total. Eight countries lack any dedicated IC, and there is an (almost total) absence of discretionary harm tests in the five main areas (with the exception of Italy which invokes these tests for documentation relation to personal affairs and commercial confidentiality). Though Germany does have the position of IC, it has none of the powers or explication of appeals process we see in countries in the eastern side of the figure.

The four countries in the north‐east quadrant are united by the presence of dedicated IC—some with binding powers and other not (Hungary and Spain). For almost all, discretionary harm tests in the five main areas (Ireland excepted which retains the right to withhold documents it judges may harm national economic competitiveness).

The south‐east quadrant has only three countries. Here, discretionary harm tests in the main areas exist (with the exception of Cyprus which has three of the five tests) combined with a dedicated IC with considerable and binding powers in all cases.

The six countries in the south‐west quadrant have discretionary harm tests in the five main areas but do not have a dedicated IC. With the exception of Austria and Belgium, appeals in these countries are made through the administrative courts and/or Ombudsman procedures.

When we lift our gaze from the specifics, and compare the countries found in each quadrant, the analysis offers some unexpected affinities. For example, in the north‐west quadrant, we find member states from different regions and different times of EU accession—Scandinavia (Sweden), Central and Eastern Europe (CEE) (Bulgaria, Czech Republic, Romania), the Baltics (Latvia, Lithuania), Southern Europe (Greece), and founding EU countries (Germany, Italy). Such diversity undermines any notions we might have about carbon copying of legislation taking place during waves of EU enlargement. Moreover, the plot also questions notions of tools based on legal families—for example, the north‐east and south‐east zones each contain mixed of civil (Croatia, Hungary, Slovenia, and Spain) and common law countries (Cyprus, Ireland, and UK).

One reason for this varied picture, and apparently unlikely affinities between countries, is the politicized nature of the development of FOI legislation. This is a high salience administrative tool whose legislative development and design is subject to intense and forensic scrutiny by a diverse range of policy actors, as shown by Worthy (2017) with reference to Britain. The result is a legislation which is not carbon copied from neighboring jurisdictions or drawn down from legal principles alone.

The key sources of variation in Ombudsman broadly confirm our expectations. PC1, explaining more than one fourth of the overall variance, puts together two choice rules and one information rule. They concern the remedial dimension and the dialectic relationship that unfolds between the Ombudsman and the public bodies to which recommendations are addressed. This is not a surprise: choice and information rules represent together more than the 53% of all Ombudsman rule types. The IGT implications are clear: the procedural aspect of Ombudsman recommendations (also including the exchange of information between the parties after the decision of the Ombudsman) is the most prominent in explaining variation in design.

The distribution of cases along PC1 confirms this intuition since it reveals the existence of the expected divide between political systems where the oversight potential of Ombudsman procedures is expressed though informality and high mutual trust between the parties (mainly old democracies) and systems where the Ombudsman is vested with quasi‐judiciary coercive prerogatives (mainly new democracies). This divide corroborates the argument about different waves of diffusion of the Ombudsman institutions (Gregory & Giddings, 2000) with late (and harder) adopters clustering in the right part of the X/Y plot.

PC2 puts together different forms of accountability. On the one hand, accountability of the Ombudsman vis‐à‐vis, not only the body that appoints her, but also the public at large. The publicity of the Ombudsman annual reports, in fact, typically brings to the fore all the cases of maladministration treated by the office before the Parliament and the public. This provides incentives for further usage by the public (Diamandouros, 2006) and constitutes a form of “name and shame” for the public bodies whose actions were reprimanded, even lacking manifest hard sanctions. The second form of accountability has to do with the boundaries of the Ombudsman jurisdiction, namely its jurisdiction over private bodies performing public functions. Clearly, countries that allow for both publicity of Ombudsman reports and coverage of private entities (upper quadrants of the X/Y plot) score strong in terms of accountability toward different positions. Finally, PC3 includes eligibility criteria to access the Ombudsman (boundary rules). Personal interest (and not time boundaries) remains a cornerstone of Ombudsman's discipline variability, as well as the incompatibility of Ombudsman investigations with judicial procedures. The IGT implications of components two and three are less neat, but note that three of the four original variables loading into these components are boundary rules, bringing us back to our expectations about the centrality of choice and boundary rules for highly proceduralized and codified instruments.

The X/Y plot of Ombudsman PCs points to low degrees of clustered convergence. Starting from the south‐west quadrant, Italy and the UK stand out. Although their designs are different, for the sake of PC1 (remedies) and PC2 (accountability) they are functionally equivalent. Italy only has regional institutions, without a central Ombudsman. In the UK, the access to the Ombudsman is filtered by MPs, the set of available remedies is limited to non‐binding recommendations and a vast number of regional/local and sectorial Ombudsman institutions exist.

Moving up (north‐west quadrant), the countries remain quite distanced with no clear clustering. Yet, among them, we find four Scandinavian and Western/Northern countries (Sweden, Denmark, Germany and the Netherlands). These countries belong to the first wave of diffusion of the Ombudsman institution and are (still) loyal to the original template: strong on accountability while drawing on informal and non‐binding remedies.

The north‐east quadrant, where accountability mechanisms are coupled with harder forms of recommendations, is the most populated with 14 countries. The two groups of countries observed in this quadrant defy classifications like waves of diffusion and legal traditions. In fact, along with new democracies (mainly grouping in the right‐end side of the plot—as noted above) we find countries like France, Austria, and Finland. The lesson we draw is that the IGT's comparative logic, aptly expressed in our analysis through orthogonal/uncorrelated components, is truly configurational. As such, one aspect/dimension of policy design highlighted by IGT may converge with existing assumptions and taxonomies, while other may allow us to detect surprising similarities in design (like in the two groups of the north‐west quadrant).

Finally, Poland, Lithuania, and Romania are in the south‐east quadrant where the hardening of Ombudsman's remedies is coupled with weak accountability mechanisms. Interestingly, these are also the only countries where the Ombudsman is allowed some form of direct sanctioning, indicating a potential (and dangerous) trade‐off between direct enforcement mechanisms and accountability rules.

## CONCLUSIONS

Our major theoretical result lies in using the IGT as lens to correctly identify the variables that matter in the design of four policy instruments (beyond the idyonsincracies of each instrument), derive measurement from theory, and deploy measurement across 28 countries to unveil suprising patterns and components that explain variation. Our challenges were to capture the structure of diverse policy instruments, avoid the pitfalls of value‐laden categories, and create regulatory indicators for cross‐country comparison. Our analysis is the first to combine the IGT with PCA to inform us about the features that matter most in explaining variation.

The IGT enabled granular analysis in three directions: we examined the distribution of rule types in the population as a whole, the IGT structure of each instrument, and the variation across 28 cases as accounted for by IGT rules. The PCAs generated coherent principal components that sharpen the IGT focus on rule types. The results often deviate from conventional comparative politics categories about civil and common law countries, varieties of capitalism, strength of pressure groups, Europeanization, and waves of accession to the EU. Actually, the empirical results have challenged and ultimately changed our priors on cross‐country variation. This, we submit, is because the comparative politics priors are suitable for macro‐comparisons. When it comes to administrative law, regulation and specific policy instruments the explanation we found is more nuanced and, as we said, granular. This new knowledge is an important contribution to the literature on regulation.

In terms of diagnostics and policy recommendations, the IGT exposes incomplete or weak designs. The institutional designs are, from an Ostromian point of view, incomplete. Specifically, across all four instruments, we lack inputs from an array of interests (aggregation) and rewards/sanctions (payoffs). Policy‐makers involved in policy reform should give serious consideration to the overall balance of institutional design across all four instruments.

Another contribution is that we considered together the four policy instruments, something that is completely original, both in the field of IGT and in the field of regulation. Future research could go even further and examine the inter‐relations and ecological relationships among the instruments. Consultation and RIA, on the one hand, and FOI and Ombudsman, on the other, have affinities. Together, the four meso action situations define a macro action situation. This article provides the foundations for scaling‐up from meso to macro. One can also build on this approach shifting to the diagnostic mode to explore how these instrument types and mixes affect socially important governance outcomes. For this, we need to combine rules‐in‐form with rules‐in‐use.

This last observation brings us to the limitations. One is that we looked at rules‐in‐form only, excluding rules‐in‐use. Another is that we did not consider the court procedures to scrutinize regulatory decisions. Additonally, we did not set up research questions with an hypothesis‐testing orientation. Our approach served us well in demonstrating the potential of the classification in terms of rule types for explanatory, hypothesis‐testing research that will take place in the future years. Future research should also include the time dimension and appraise the role of the EU institutions in shaping policy design over time, especially in terms of waves of accession.

## Supporting information

Supplementary Material

## ENDNOTES

1. Rule types are conceived, among other reasons “to understand how action situations are constructed, […] to cope with the immense diversity of rules by clustering them into seven generic rules and […] to study the underlying universality of rules” (Ostrom, 2005, p. 175).

2.
https://www.theioi.org/the‐i‐o‐i.

3. The judicialization of the Ombudsman office may also reflects an objective weakness of the judiciary branch in Central and Eastern European countries (Gregory and Giddings, 2000).

4. More detail on the data collection are presented in Section 1 of the Appendix.

5. This population is diverse in terms of administrative law traditions, efficiency of public administration, waves of Europeanization, models of capitalism and institutional change—with the Eastern European institutions having experienced the change from communist rule to democracy.

6. We should be clear that despite using the UK label, the legislation coded for this study is the FOI legislation for England, Wales and Northern Ireland Freedom of Information Act 2000). Owing to its distinct legal system (combination of common and civil law) public authorities in Scotland are covered by separate legislation though it is similar (Freedom of Information [Scotland] Act 2002).

## References

[psj12440-bib-0001] Banisar, D. 2006. Freedom of Information around the World. Privacy International.

[psj12440-bib-0002] Blanke, H.‐J. , and R. Ricardo Perlingeiro . 2018. “Essentials of the Right of Access to Public Information: An Introduction.” In The Right of Access to Public Information, edited by H.‐J. Blanke and R. Perlingeiro , 1–68. Berlin: Springer.

[psj12440-bib-0003] Bozzini, E. , and S. Smismans . 2016. “More Inclusive European Governance Through Impact Assessments?” Comparative European Politics 14 (1): 89–106.

[psj12440-bib-0004] Bunea, A. 2016. “Designing Stakeholder Consultations: Reinforcing or Alleviating Bias in the European Union System of Governance?” European Journal of Political Research 56 (1): 46–69.

[psj12440-bib-0005] Carter, D. P. , C. M. Weible , S. N. Siddiki , J. Brett , and S. M. Chonaiew 2015. “Assessing Policy Divergence: How to Investigate the Differences between Law and a Corresponding Regulation.” Public Administration 93 (1): 159–76.

[psj12440-bib-0006] Carter, D. P. , C. M. Weible , S. N. Siddiki , and X. Basurto . 2016. “Integrating Core Concepts from the Institutional Analysis and Development Framework for the Systematic Analysis of Policy Designs: An Illustration from the U.S. National Organic Farming Regulation.” Journal of Theoretical Politics 28 (1): 159–85.

[psj12440-bib-0008] Crawford, S. E. S. , and E. Ostrom . 1995. “A Grammar of Institutions.” American Political Science Review 89 (3): 582–600.

[psj12440-bib-0009] De Francesco, F. 2013. Transnational Policy Innovation: The OECD and the Diffusion of Regulatory Impact Analysis. Colchester: ECPR Press.

[psj12440-bib-0010] Diamandouros, P. N. 2006. “The Ombudsman Institution and the Quality of Democracy.” Lecture by the European Ombudsman at the Centre for the Study of Political Change, University of Siena, Siena, Italy, 17 October 2006.

[psj12440-bib-0011] Dragos, D. C. , P. Kovač , and A. T. Marseille . 2019. “From the Editors: The Story of a Data‐Driven Comparative Legal Research Project on FOIA Implementation in Europe.” In The Laws of Transparency in Action, edited by D. C. Dragos , P. Kovač , and A. T. Marseille , 1–7. Basingstoke: Palgrave.

[psj12440-bib-0012] Dunlop, C. A. , J. C. Kamkhaji , and C. M. Radaelli . 2019. “A Sleeping Giant Awakes? The Rise of the Institutional Grammar Tool (IGT) in Policy Research.” Journal of Chinese Governance 4 (2): 163–80.

[psj12440-bib-0013] Dunlop, C. A. , J. C. Kamkhaji , C. M. Radaelli , G. Taffoni , and C. Wagemann . 2020. “Does Consultation Count for Corruption?” Journal of European Public Policy 27 (11): 1718–41.

[psj12440-bib-0014] Espinosa, S. 2015. “Unveiling the Features of a Regulatory System: The Institutional Grammar of Tobacco Legislation in Mexico.” International Journal of Public Administration 38 (9): 616–31.

[psj12440-bib-0015] Gregory, R. , and P. J. Giddings , eds. 2000. Righting Wrongs: The Ombudsman in Six Continents. Vol. 13. IOS Press.

[psj12440-bib-0016] Knill, C. , K. Schulze , and J. Tosun . 2012. “Regulatory Policy Outputs and Impacts: Exploring a Complex Relationship.” Regulation & Governance 6 (4): 427–44.

[psj12440-bib-0018] Listorti, G. , E. B. Ferrari , S. Acs , G. Munda , E. Rosenbaum , P. Paruolo , and P. Smits . 2019. The Debate on the EU Better Regulation Agenda: A Literature Review. Brussels: Publications Office of the European Union.

[psj12440-bib-0019] Maggetti, M. , F. Gilardi , and C. M. Radaelli . 2012. Designing Research in the Social Sciences. London: Sage.

[psj12440-bib-0020] McGinnis, M. D. 2011. “Networks of Adjacent Action Situations in Polycentric Governance.” Policy Studies Journal 39 (1): 51–78.

[psj12440-bib-0021] Mendel, T. 2008. Freedom of Information: A Comparative Legal Survey. 2nd ed. Paris: UNESCO.

[psj12440-bib-0022] Möck, M. , C. S. Vogeler , N. C. Bandelow , and B. Schröder . 2020. “Layering Action Situations to Integrate Spatial Scales, Resource Linkages, and Change over Time: The Case of Groundwater Management in Agricultural Hubs in Germany.” Policy Studies Journal. 10.1111/psj.12377

[psj12440-bib-0023] Muscar, N. P. , and B. Cottier . 2017. Comparative Study of Different Appeal and Control National Mechanisms Regarding Access to Public Information in Six Council of Europe Member States. Brussels: Council of Europe.

[psj12440-bib-0024] OCED . 2011. Government at a Glance: Transparency in Government. Paris: OECD Publications.

[psj12440-bib-0025] OECD . 2019. Better Regulation Practices Across the European Union. Paris: OECD Publications.

[psj12440-bib-0027] Ostrom, E. 2005. Understanding Institutional Diversity. Princeton, NJ: Princeton University Press.

[psj12440-bib-0028] Ostrom, E. 2007. “Institutional Rational Choice: An Assessment of the Institutional Analysis and Development Framework.” In Theories of the Policy Process, edited by P. A. Sabatier , 2nd ed., 21–64. Boulder, CO: Westview Press.

[psj12440-bib-0029] Ostrom, E. 2011. “Background on the Institutional Analysis and Development Framework.” Policy Studies Journal 39 (1): 7–27.

[psj12440-bib-0032] Radaelli, C. M. 2005. “Diffusion Without Convergence: How Political Context Shapes the Adoption of Regulatory Impact Assessment.” Journal of European Public Policy 12 (5): 924–43.

[psj12440-bib-0033] Radaelli, C. M. 2009. “Measuring Policy Learning: Regulatory Impact Assessment in Europe.” Journal of European Public Policy 16 (8): 1145–64.

[psj12440-bib-0034] Radaelli, C. M. 2010a. “Regulating Rule‐Making via Impact Assessment.” Governance 23 (1): 89–108.

[psj12440-bib-0035] Radaelli, C. M. 2010b. “Rationality, Power, Management and Symbols: Four Images of Regulatory Impact Assessment.” Scandinavian Political Studies 33 (2): 164–88.

[psj12440-bib-0036] Radaelli, C. M. 2020. “Regulatory Indicators in the European Union and the Organization for Economic Cooperation and Development: Performance Assessment, Organizational Processes, and Learning.” Public Policy and Administration 35 (3): 227–46.

[psj12440-bib-0037] Schlager, E. , and M. Cox . 2017. “The IAD Framework and the SES Framework.” In Theories of the Policy Process, edited by C. M. Weible and P. A. Sabatier , 4th ed. Boulder, CO: Westview Press.

[psj12440-bib-0038] Siddiki, S. N. , T. Heikkila , C. M. Weible , R. Pacheo‐Vega , D. Carter , C. Curley , A. Deslatte , and A. Bennett . 2020. “Institutional Analysis with the Institutional Grammar.” Policy Studies Journal. 10.1111/psj.12361

[psj12440-bib-0039] Siddiki, S. N. , C. M. Weible , X. Basurto , and J. Calanni . 2011. “Dissecting Policy Designs: An Application of the Institutional Grammar Tool.” Policy Studies Journal 39 (1): 79–103.

[psj12440-bib-0041] Strauss, P. L. , T. T. Smith , and L. Bergkamp . 2008. Administrative Law of the European Union: Rulemaking, Chicago, IL: American Bar Association.

[psj12440-bib-0042] UNESCO . 2016. UNESCO and International Day for Universal Access to Information, Paris: UNESCO.

[psj12440-bib-0043] Wiener, J. B. 2007. “Better Regulation in Europe.” Current Legal Problems 59 (1): 447–518.

[psj12440-bib-0044] World Bank . 2015. Strengthening the Regulatory Impact Assessment Framework in Romania. AllioǀRodrigo Consulting. Final Report, September.

[psj12440-bib-0045] Worthy, B. 2017. The Politics of Freedom of Information. Manchester: Manchester University Press.

