# Supplementary material for: Measuring design diversity: A new application of Ostrom's rule types
Source: Policy Stud J. 2021 Jul 20;50(2):432–52. doi: 10.1111/psj.12440 (PMC9292763; doi:10.1111/psj.12440)
Supplement: Supplementary file 1 — Supplementary Material [file PSJ-50-432-s001.docx]

**Appendix**

This appendix has three aims. First, to provide a detailed account of how we collected and coded the data and resulting the four datasets (Section 1). Second, to provide technical information on the Principal Component Analyses (PCAs) (Section 2). Third, to describe the Principal Components (PCs). Additional material about the PCAs is available upon request.

**Section 1**

In this section, we offer the reader some more insight into the data collection exercise that led to the creation of the four datasets utilized in the article.

The four rulemaking procedures under investigation are mapped and measured through an innovative and theory-informed data collection methodology – Institutional Grammar Tool (IGT) rule types (as explained in the main body of the text). This approach can be systematically replicated as it draws on primary and secondary legal texts. It differs from subjective measures based on expert opinions.

To achieve the IGT-informed typological approach to measurement we worked with a team of 40 administrative lawyers. For each of the EU-28 countries we identified the legal bases of the four instruments in force as of June 2018 (grounded in hard law or on soft guidance documents) and retrieved text in original language and in English translation. Relevant portions of legal texts were gathered using a protocol based on Ostrom’s rule categories (more on this below). Thus, when considering the guidance and/or law on consultation for country X we retrieved the exact text (articles, clauses, or entire sections) where positions are defined, boundaries set, information flows described, choice prescribed, and so on. As a result, our data points are sentences extracted from relevant legal bases.

To achieve this, we created a data collection protocol composed of four tasks – the same for all the instruments. We then launched a public call for experts across the EU with the aim of finding native speaking legal experts who could collect legal texts in original language and translating them in English.

Under Task 1 of the protocol (the same for all the EU 28 countries), we required our experts to detect the relevant legal base(s) for the four instruments in each country, including year of adoption and most recent amendments. Under Task 2, the bulk of our protocol, we required to extract all the statements included in the legal base(s) and collect them according to the seven rule types. To carry out this task, we relied on clearly written instructions further explained in the context of a webinar, a face-to-face workshop and plus a number of one-to-one sessions via Skype. Task 3 of the protocol required the expert to draw a flowchart graphically summarising the key steps of the procedure of interest. Task 4 revolved around a series of open-ended questions.

Once we received the 112 (28 x 4) protocols, we validated the data in our team, with the additional guidance of the senior administrative lawyers who sit on the International Advisory Team of the project. This is because the data collectors we hired are recognized legal experts, but we cannot expect that their understanding of Ostrom rule types led to a flawless categorization. Working in pairs to increase the reliability of the categorisation, we validated all the protocols (i.e. reallocated the extracted statements in the correct Ostrom categories). In some cases, we went back to the lawyers asking precise, factual questions regarding the legal bases.

Once we had all protocols validated, we created the four data architectures including:

_33 rules/variables for Consultation
_45 rules/variables for RIA
_64 rules/variables for FOI
_61 rules/variables for Ombudsman procedures

These are mainly “Yes” or “No” micro-procedural items which reflect the rules extracted from the legal bases – they are summarized in Table 2 in the manuscript. In practice, we transformed our textual data points into mainly dichotomous variables indicating the presence/absence of a given rule.

Along with variables based on Ostrom rule types we also collected and included in the data architectures a small number of background variables detailing, for instance, the presence of a hard as opposed to soft legal base or the regional/sectorial coverage of the instruments. These rules were used in the PCAs.

**Section 2**

We employed PCA over alternative types of analysis for a series of reasons. First, the choice of PCA reflects an inductive approach towards our data. In other words, we chose a dimension reduction technique that enables us to summarise our data without reference to a specific model or latent/hidden traits of the data.^[[1]](#footnote-1)^ The second reason is conceptual: we wanted a dimension reduction technique based upon the analysis of correlations. This is because we aim at exploring the internal structure of the data with particular regard to the correlations between and across Ostrom’s rule types. PCA allows us to do so in an exploratory fashion and without having to draw on the restrictive assumptions that characterize Confirmatory Factor Analysis and to an extent also Exploratory Factor Analysis.^[[2]](#footnote-2)^ PCA is also assumption-free and robust in terms of type of variables utilized and their distributional features (Jolliffe 2002), a plus that does not apply to Multiple Correspondence Analysis (MCA). Moreover, according to several scholars, with datasets based on dichotomous items, PCA and MCA may be equivalent, with the former being the more general form.^[[3]](#footnote-3)^ Another beneficial feature of PCA is that, when a varimax rotation is performed, it allows, by maximising the overall variance, to detect and hierarchically order those components which represent the major sources of variation within the datasets. In our parlance, we refer to these sources as “difference-making conditions”. Furthermore, these sources of variation represented by the components are orthogonal, i.e. uncorrelated, facilitating successive analysis and interpretation.

We draw on the seminal treatment of Jolliffe (2002) and on more recent scholarship (Abdi and Williams 2010; Shaukat et al. 2016) to argue that PCA:

_Can be utilized on a set of (mainly) dichotomous variables (Jolliffe 2002, 339-340);

_Can be meaningfully utilized on relatively small samples (Shaukat et al. 2016);

_Can be deployed to summarize complex information conveyed by a dataset when Principal Components are conceptually interpretable (Abdi and Williams 2010).

We now elaborate more specifically on the low-N limitation, as suggested by an anonymous reviewer. The theme of investigating PCA and related techniques’ suitability to High Dimension, Low Sample Size (HDLSS) data is widely discussed in the methodological literature (and, to be a honest, to a level of technicality which does not belong to the rather simple structure of the data we analyzed). Further examples besides those already cited above are Hwang et al. (2002) and Jung and Marron (2009).

In short, a number of technical publications specifically on PCA highlight that rules of thumb for sample size may work well for ANOVA or regression settings but not so much for PCA or factor analysis. This is because rules of thumb are, by their very nature, not case-specific (in this case, database-specific) and hence are insensitive with respect to the nature/structure of the data. To illustrate, whereas a big sample size is required to extract meaningful components when manifest variables are mainly uncorrelated, this is not the case when the variables are highly correlated. The main conclusion of the literature is hence that low sample sizes are acceptable and valid if the aim is descriptive and the directionality of the data is conceptually coherent. We draw on the seminal treatment of Jolliffe: “In any case, a description of the sample, rather than inference about the underlying population, is often what is required, and the PCs describe the major directions of variation within a sample, regardless of the sample size.” (Jolliffe 2002, 68).

Coming to our data, the number of observations (28) happens to be lower than the number of manifest variables which characterize each dataset. As already said, though, drawing on Shaukat et al. (2016) and other older contributions, we reject the notion that small-n datasets are unsuited to PCA. To verify this against our data, when performing the PCAs, we looked carefully into measure of sample adequacy (for imperfect they are). We report them below.

Table A1. Measures of sample adequacy

| Instrument | Kaiser-Meyer-Olkin (KMO) measure of sample adequacy  (rule of thumb >.6) | Bartlett’s test of sphericity (rule of thumb <.05) |
| --- | --- | --- |
| Consultation | .444 | .003 |
| RIA | .458 | .000 |
| FOI | NA | NA |
| Ombudsman (OM) | .454 | .000 |

Three out of the four PCAs (Consultation, RIA and OM) reach sample adequacy according to the Bartlett’s test of sphericity. This allows us to reject the null hypothesis of being working on identity matrixes. On the other hand, the results of the KMO indicator are less encouraging as the measures slightly fall short of the threshold suggested by the literature (>.6). When it comes to FOI, the two measures cannot be calculated as the correlation matrix is not positive definite.

Yet, beyond statistical tests, another important way to assess the adequacy and validity of PCAs is conceptual. It entails controlling *ex post* whether the extracted components are grouping variables that are measuring the same construct. Indeed, we can confidently claim that all components across the four instruments do so (see Table 3 in the text), with the FOI PCA being perhaps the neatest in this regard and the other being more than satisfactory in terms of interpretability. Furthermore, the FOI’s components exhibit a simple overall structure which occurs when “each component has a small number of large loadings and a large number of zero (or small) loadings” (Abdi and Williams 2010, 442). In other words, simple structures can be observed when a subset of conceptually related manifest variables significantly load on separate individual components, thereby confirming that these manifest variables did indeed measure the same construct which is now aptly summarized by the orthogonal components. As already argued, the components extracted from the four datasets (FOI in particular) all show this simple structure since they are easily interpretable and conceptually meaningful and group variables which have a strong logical consistency. Moreover, they single-out those sources of variation that were detected by qualitative literature. This is of utmost importance because, differently from extant contributions, we have not organized our data collection around those sources or any specific model but rather according to Ostrom rule types. The fact that PCAs extracted coherent principal components is both solid proof of the conceptual (albeit not technical) validity of the PCAs and an important result in terms of aligning the Ostromian approach through which we have produced our data with the indications of non-Ostromian literature. Thus, in our view, and this is also reinforced by the literature cited above, our PCAs are all fully valid.

To conclude, the small sample size is certainly a limitation of this study, but we would and do not term it as a significant one granted that three out of four PCAs are technically adequate according to a major measure of adequacy of the data structure and all of them are conceptually consistent.

The main limitation of small-N PCAs is that it may not allow to perform a fully efficient dimensional reduction (but this, as previously noted, has also to do with the bivariate correlations observed among manifest variables). When the sample size increases, in fact, rotation typically allows for few PCs characterized by high eigenvalues and high degrees of explained variance. In practical terms, when observations greatly outnumber manifest variables (e.g. a subject to item ratio of 10 to 1) few components are normally enough to account for large portions of variance, providing for a highly efficient dimensional reduction. In these cases, normally, the first two components are enough to explain almost all the structural variance observed in the dataset. In our case, and this is the main technical limitation, more components are needed to account for structural variance (let us recall that components with eigenvalues < 1 are considered irrelevant as they account for noise variance). As a result of this limitation, in presenting the PCs we decided to stick to a conservative criterion whereby the studied components explained at least 50% of the overall variance (the criterion is conservative but present in the literature, see: Jolliffe, 2002; Fritsch, Kamkhaji and Radaelli, 2017). Taking into account a share of noise variance ranging from 20 to 30% in each of the datasets, this means that some 25% of the total variance remains unexplained if we look only at the first two or three components. Hence, we maintain, supported by both technical and conceptual considerations and by the literature, that the PCAs we performed are fully valid but we acknowledge that to account for all the structural variance captured by PCA we need to look into more than the first two or three PCs. For reasons of space and clarity this is not feasible in the context of a single article presenting four different PCAs, hence we decided to present and discuss only those components which allow to explain more than 50% of the overall variance. We think that this is suited for the type of exploratory, descriptive analysis we perform in the article but we are aware that when we will use the PCAs to create indicators related to the four tools we will have to use more PCs and PC scores to come up with robust measures.

To conclude, a few remarks on the practical computation of the PCAs and on the strategies employed to guarantee robustness are in order.

In the first step of each of the four PCAs, we analysed the bivariate correlation matrixes looking for a number of significant correlations, the prerequisite to carry out a PCA on the correlation matrix. The result was, for all the four datasets, that a sufficient number of bivariate correlations between rules (i.e. initial manifest variables) existed, were statistically significant (one-tailed significance, p<.05) and sizeable (i.e. above the .3 threshold), corroborating the choice of running PCAs based on the correlation matrixes.

Having already discussed the issue of sample adequacy, we move to the iterative steps implemented to come up with robust PCAs. These steps are about the successive elimination of non-relevant/redundant variables form the analysis and are the following:

_Removal of all variables which show a communality <.5. The communality is the share - expressed on a 0-1 scale – of the variance of each variable that may be explained by the extraction of the principal components. If the coefficient was below .5, we removed the variable and reran the PCA without it.

_Removal of those variables which show a complex structure. A complex structure can be observed when a variable shows a significant loading – i.e. >0.4 – on more than one component. When we identified such a variable, we removed it from the set of manifest variables and reran the PCA without it.

We adopted a very simple criterion for the retention of components for further analysis and discussion. We stick to the criterion of more than 50% of explained variance (Jolliffe 2002). This threshold is reached with the first two components for Consultation and with the first three for the other instruments.

Finally, plotting cases along the first two components, requires the computation of PC scores. To this end we employed the most common of the “refined methods”, that is, regression scores. The latter were standardized to range from 0 to 1 to improve interpretability.

**Section 3 - The Principal Components**

We now elaborate on the nature of the components in terms of loading manifest variables and their typology. As argued above when discussing the validity of the four PCAs, a key element to adjudicate whether a PCA is adequate and robust is to look into the first Principal Components and evaluate whether they show a simple structure, which is also interpretable. This kind of assessment is also instrumental in naming the components, that is, in finding a conceptual interpretation of what the newly computed variables (i.e. the components, which are a mix of manifest variables) mean in practice. As a reminder, a simple structure emerges when a component is characterised by few manifest variables significantly loading on it and the other showing a low/close to zero loading coefficient. Technically, a simple structure emerges from PCA when “each component has a small number of large loadings and a large number of zero (or small) loadings” (Abdi and Williams 2010, 442). Therefore, simple structures can be observed when a subset of conceptually related manifest variables significantly load on a single component, thereby confirming that these manifest variables did indeed measure the same construct which is now aptly summarized by the component. This is indeed the case for our PCAs (see Table 3 in the main text). The extracted PCs are both technically sound (i.e. they are characterized by few variables showing a significant loading and the other showing a small/close to zero loading) and conceptually consistent (i.e. they are loaded by a coherent subset of manifest variables which taken together measure a relevant latent construct within the data – something that allowed us to name the components according to key overarching dimensions of the four instruments).

Focusing on the latter aspect, which allows us to discuss and elaborate on the Ostromian features of the individual components, conceptual consistency is achieved when the manifest variables loading on a component are coherent and, taken together, represent a key discrete (recall that the PCs are orthogonal) dimension of the phenomenon. As it can be appreciated from Table 3, out of 11 Principal Components retained for the analysis of the four instruments, 6 components are loaded by variables which belong to a single Ostrom category. They are:

_Component 2 of N&C (Scope rules)

_Components 1 (Boundary rules) and 2 (Choice rules) of RIA

_Components 2 and 3 of FOI (Boundary rules for both)

_Component 3 of Ombudsman (Boundary rules)

These components have the benefit of being easy to interpret further confirming the validity of the PCAs in reducing complexity in a consistent fashion. Beyond that, the fact that rules belonging to the same typology load on individual components further attests the configurational nature of rule types within action situations and the usefulness of using true types to unearth the structural dimensions of policy interactions. Looking at them individually, PC2 of N&C owes its name directly to the Ostromian category and shows the importance of stating goals and setting objectives in the context of institutional texts such as guidelines (which constitute the typical legal base of consultation procedures across our sample).

Regarding RIA’s first two components, the fact that they are constituted, respectively, of Boundary and Choice rules document two phenomena which are widely discussed in the specialised literature. The first is that the use of evidence-based instruments may be trumped by the presence of “escape clauses” which (conveniently) insulate decision-making from expert input and public scrutiny. The second is that an instrument like RIA is naturally geared toward a utilitarian approach which consistently involves, not only the quantitative computation of benefits related to policy options, but also the regulatory/market failure (measured through the analysis of the status quo) as starting point of decision-making. The advancement of recasting these design aspects of RIA in terms of conceptual, theoretical categories is not simply taxonomical but it surely extends to the territory of practice and (regulatory) reform.

The PCs of FOI also show interesting structural features. It is essential to notice how PC2 and PC3 carefully account for the importance of exclusion clauses when it comes to the access to public information. Interestingly, Ostromian boundaries are shown to extend beyond strict actors’ eligibility and investing also the discretion enjoyed by the positions held by public actors (regardless whether this position is held by an IC or by individual articulations of the PA).

Turning to the Ombudsman, component 3 (loaded by two Boundary rules) goes in the same direction of the PC2 and PC3 of the FOI (extended meaning of boundaries) but perhaps more interesting is the configuration of PC1 about “Remedies”. Interestingly, the component is loaded by two Choice rules about, indeed, remedies and one Information rule which is anyhow connected to the compliance with remedies set by the Choice rules. Like in PC1 of FOI about the Information Commissioner, we see different rule types connected to a structural dimension of the procedure (remedies for the Ombudsman and the Information Commissioner for FOI) clustering together as they measure an underlying construct which transcends Ostrom categories and is strongly case-specific.

Overall, from the analysis of the Ostromian content of the components we learn that rule types are effective analytical categories for our sample. In fact, they can work:

_in a discrete fashion (i.e. rules belonging to a single rule type loading onto a single component) rendering hence latent dimensions of the analysed procedure which are directly connected to Ostrom typologies;

_in a configurational fashion (i.e. different rule types loading onto individual components) showing to be able to let other structural dimensions of the design emerge.

**References**

Abdi, H. and L.J. Williams (2010) “Principal component analysis”, *WIREs Comp Stat*, 2: 433-459.

Fritsch, O., Kamkhaji, J.C. and C.M. Radaelli (2017) “Explaining the content of impact assessment in the United Kingdom: Learning across time, sectors, and departments”, *Regulation & Governance*, 11(4): 325-342.

Gower, J.C. 1966. “Some distance properties of latent root and vectormethods used in multivariate analysis”, *Biometrika*, 53: 325–338.

Hwang, D., Schmitt, W.A., Stephanopoulos, G. and G. Stephanopoulos (2002) “Determination of minimum sample size and discriminatory expression patterns in microarray data”, *Bioinformatics,* 18(9): 1184-93.

Jolliffe, I.T. (2002) *Principal Component Analysis*. New York: Springer.

Jung, S. and J.S. Marron (2009) “PCA consistency in high dimension, low sample size context”, *Ann. Statist.,* 37 (2009), no. 6B, 4104—4130.

Shaukat, S., Rao, T., and M.A. Khan (2016) “Impact of sample size on principal component analysis ordination of an environmental data set: effects on eigenstructure”, Ekológia, 35(2): 173-190.

**Websites**

<https://stats.stackexchange.com/questions/34870/about-the-usage-of-catpca/34878#34878>

1. “[F]actor analysis attempts to achieve a reduction from *p* [manifest variables] to *m* dimensions by invoking a *model* relating $x_{1}, x_{2},\ldots, x_{p}$ to *m* hypothetical or latent variables […] PCA differs from factor analysis in having *no explicit model*.” (Jolliffe 2002, p. 151, emphasis in original). [↑](#footnote-ref-1)
2. Furthermore, analysing the correlation matrix for multicollinearity would be in order in the context of Confirmatory or Exploratory Factor Analysis. However, because we are dealing with a PCA based on orthogonal varimax rotation, multicollinearity is not an issue. [↑](#footnote-ref-2)
3. “The idea of correspondence analysis as a form of PCA for nominal data is valid for any number of binary variables.” (Jolliffe 2002, 343). See also Gower (1966) and <https://stats.stackexchange.com/questions/34870/about-the-usage-of-catpca/34878#34878> [↑](#footnote-ref-3)
